# A Text-Book of the Theory and Practice of Medicine

**Published:** 1894-03

**Authors:** 


### Reviews of Books.

*A Text-book of the Theory and Practice of Medicine.* By American Teachers. Edited by William Pepper, M.D. Vol. I. Pp. xii., 909. Philadelphia: W. B. Saunders. 1893.

Thirteen teachers of practical medicine in the leading schools of America have associated themselves in the production of a volume which represents truly the best teaching of the science and art of medicine at the present time. They have given us nearly a thousand pages, in the best of type, with 58 figures and three large plates, but worthy of a better binding. It is difficult to criticise a volume of this kind, in which eminent specialists express their opinions with authority. The reviewer can only sit at their feet and learn.

The first article is by Dr. J. S. Billings, on the subject of

"Hygiene," including disinfection, isolation, and other principles of preventive medicine.

Dr. William Pepper's article on "Typhoid Fever" extends to 81 pages, and is one of the most complete accounts on record; but we venture to think that phenacetin deserves to be dissociated from antipyrine and acetanilide, and to be given more freely and vigorously. Of the intestinal antiseptics preference is given to nitrate of silver, which in doses of one-fourth of a grain is considered to be a useful part of the regular treatment of typhoid. Turpentine, as a powerful antiseptic and a good stimulant, is believed to be of unquestionable value, whilst calomel in purgative doses is totally objected to. Various intestinal antiseptics are mentioned as having been advocated, but carbolic acid is not one of them; and the writer, appearing to have had little experience of any, is a little incredulous as to their value.

The various exanthems are well described by Dr. James J. Whittaker, who declines to mention antiseptic inunction in scarlet fever, and states that the treatment is wholly symptomatic.

Dr. Gilman Thompson describes diphtheria, which he treats as an acute, infectious, inoculable disease, developing a local lesion at some period of its course; but only in exceptional cases does the local lesion first appear. Further researches appear to have shown that the local focus is the disease itself, and that inoculation of the system as a whole need not occur if the local lesion be recognised and treated with vigour.

Dr. Horatio C. Wood's articles on "Various Diseases of the Nervous System," and those of Dr. Wm. Osler on "Diseases of the Spinal Cord, the Nerves, and the Muscles," form a fairly complete and trustworthy outline of our available knowledge of the topics with which they deal.

We know no text-book in the English language which could be spoken of as better than the first of the two volumes which are to comprise this work.

